# Development and Implementation of a National Programme for the Management of Severe and Very Severe Pneumonia in Children in Malawi

**DOI:** 10.1371/journal.pmed.1000137

**Published:** 2009-11-10

**Authors:** Penelope Marjorie Enarson, Robert Gie, Donald A. Enarson, Charles Mwansambo

**Affiliations:** 1 International Union Against Tuberculosis and Lung Disease, Child Lung Health Division, Paris, France; 2 Department of Paediatrics and Child Health, Faculty of Health Sciences, Stellenbosch University, Tygerberg, South Africa; 3 Kamuzu Central Hospital, Department of Paediatrics, Lilongwe, Malawi

## Abstract

Penelope Enarson and colleagues describe the development, scale-up, and achievements of a national pneumonia program in Malawi, which is based on a successful anti-tuberculosis service delivery model.

**Summary Points**

- More than 20% of child mortality in poor countries is due to pneumonia.
- Standard case management of pneumonia has the potential to reduce overall child mortality, provided it can be delivered effectively.
- The government of Malawi recently introduced a national programme for the delivery of standard case management for pneumonia in children in Malawi with the help of technical and donor partners.
- This Health in Action article describes the development, scale-up, and achievements of this national programme, which is based on a successful antituberculosis service delivery model, and discusses the challenges facing the implementation of this adapted service delivery model.

## Background

The reduction of child mortality by two-thirds from its 1990 level by 2015—the fourth United Nations Millennium Development Goal—is a major challenge. Pneumonia accounts for much (≥20%) of this mortality in poor countries, but standard case management (SCM) of pneumonia [1] has the potential to reduce overall child mortality. A recent meta-analysis estimated that SCM of pneumonia could reduce overall mortality in neonates, infants under 1 y old, and children aged 0–4 y, respectively, by 27%, 20%, and 24%, and pneumonia-specific mortality by 42%, 36%, and 36% in the same age groups [2].

However, even proven intervention strategies cannot function without an effective “delivery strategy” [3]. For, example, although the World Health Organization (WHO)/United Nations Children's Fund has developed an Integrated Management of Childhood Illness (IMCI) strategy to reduce child mortality, of the 100+ low- and middle-income countries that introduced IMCI in the 1990s, only 48% had scaled up coverage by the end of 2002. Weak health systems were the main cause of this failure with the poorest countries doing worst [3].

We describe here the development and scaling-up of a country-wide delivery strategy of SCM for pneumonia in children in Malawi, a country where more than 200 children per thousand die before they are 5 y old.

## The Health Service Delivery Model

The International Union Against Tuberculosis and Lung Disease (The Union) previously pioneered an effective delivery model for antituberculosis services [4] for patients in poor countries. The approach used in this framework, which is one of the most cost-effective health interventions [5] devised so far, was incorporated into the WHO Stop TB Strategy and, by 2005, it had been successfully introduced into 190 countries [6]. Its principles include political commitment, standardized diagnosis and treatment, training, logistics, recording and reporting, supervision, and evaluation of services.

The Union has adapted this model to improve the management of severe and very severe pneumonia in children admitted to first-level (district) hospitals, institutions that are accessible to the whole population but where care is often deficient [7]–[9]. The framework allows accurate accounting of services, materials, and training, facilitates the calculation of outcome per unit of cost, and permits the management of supplies to avoid disruption of essential materials.

The model focuses on strengthening district hospitals and their associated health centres—the basic management unit. Thus, it should facilitate the management of pneumonia in institutions that are peripheral enough to promote access but central enough to facilitate monitoring and evaluation.

The core elements of this approach for the delivery of SCM for pneumonia are:

- Political commitment of the government for countrywide implementation within existing health systems and the support of a donor partner to assist until the programme is self-sustaining. This element implies that there is/are:
  - an existing structure for delivering the services;
  - access for all patients to the services;
  - financial resources to sustain activities.
- Diagnosis and treatment based upon SCM (Table 1) with a system of quality control.
- Training of clinical staff in SCM.
- Logistics to purchase and distribute uninterrupted supplies of standardized drugs.
- Recording and reporting of clinical outcomes of pneumonia.
- Supervision and evaluation of the services.

**Table 1:** Pneumonia SCM.

Diagnosis	Presenting Signs and Symptoms	Recommended Treatment Regimens
**Very severe pneumonia**	Respiratory rate: ≥50 aged 2–11 mo; ≥40 aged 12–59 mo; Lower chest wall in-drawing; Cyanosis; unable to drink; reduced level of consciousness; grunting (infants)	Chloramphenicol 25 mg/kg 8 hourly; or ampicillin 50 mg/kg IM four times daily plus gentamicin 7.5 mg/kg once daily; or if ampicillin unavailable, benzylpenicillin 50,000 units/kg every 6 h plus gentamicin 10 d antibiotic treatment
**Severe pneumonia**	Respiratory rate: ≥50 aged 2–11 mo; ≥40 aged 12–59 mo; lower chest wall in-drawing	Benzylpenicillin 50,000 units/kg every 6 h; or if injectable penicillin unavailable or referral is not possible, then oral amoxicillin 25 mg/kg twice daily for 5 d
**Nonsevere pneumonia**	Respiratory rate: ≥50 aged 2–11 mo; ≥40 aged 12–59 mo	Cotrimoxazole (trimethoprim 4 mg/kg–sulphamethoxazole 20 mg/kg) twice daily for 5 d; or amoxicillin 25 mg/kg twice daily 3 d antibiotic treatment

IM, intramuscularly; SCM, child aged 2–59 mo.

## Implementation of a Child Lung Health Programme

In 1999 the Government of Malawi asked The Union to assist it in the development and implementation of a Child Lung Health Programme (CLHP) to manage children under 5 y old hospitalized with severe/very severe pneumonia. The government identified the following problems: (1) inadequate health-worker skills in district hospitals; (2) inadequate supplies of antibiotics and equipment to administer oxygen therapy; (3) deficient use of strategic information.

The resultant CLHP was incorporated into the existing paediatric wards and outpatient departments in first-level government hospitals and implemented solely by the personnel within these services. It was coordinated with existing policies for the treatment of acute respiratory infections (ARI) and for the implementation of the IMCI strategy and centrally managed by the ARI/IMCI team.

As part of the existing health system [6], the national Ministry of Health (MOH) contributed 70.8% of the CLHP's running costs; these costs paid for facilities and human resources. The donor (The Bill and Melinda Gates Foundation) provided the remaining costs (a total of US$1.93 million over a 6-y period); 21% of these costs were used for investment and 79% covered operating costs.

CLHP implementation was carried out in four “work-packages” that identified activities for each year and set a framework for preparing budgets (see Table 2). Implementation across the country was done step-wise to ensure that the policies were adapted to local circumstances; expansion used previously developed sites as training facilities.

**Table 2:** Key activities within each work package.

Coordination	Training	Monitoring/Evaluation	Supply
Planning/budgeting; training/supervision; recording/reporting; supply management; sSectoral coordination^a^: define sectoral stakeholders; develop sectoral mechanisms; define stakeholders in other sectors; outline mechanism for inter-agency coordination	Standard operating manuals development; training materials development; training courses; training follow-up; supervision; describe frequency by level^b^; define procedures; standardize forms; outline reporting mechanisms	Describe frequency by level^b^; define procedures; develop standardized forms; outline reporting mechanisms; analyze progress toward targets; prepare routine reports; establish peer review processes; establish quality assurance processes	Outline core requirements; define procurement plan; describe logistics system; monitor order/delivery; inspect/audit stock

a Coordination of CLHP with other sectors involved in health services.

b The frequency of training and monitoring/evaluation of regions and districts by more central bodies.

## The CLHP in More Detail

### Signing a Formal Agreement

In 2000, The Union and the Government of Malawi signed a formal agreement outlining the government's commitment to the CHLP [10].

### Situation Analysis

A standardized evaluation of the epidemiology of childhood lung disease, the nature of the health services available to deal with them, and the efficiency, equity, and accessibility of these services was carried out [11].

### Developing a Plan of Action

A 5-y plan designed to achieve countrywide CLHP coverage was prepared on the basis of available documents and the situation analysis. The plan included a manual of policies and procedures, a calendar of activities, a budget, a description of the responsibilities of the parties involved in implementation, an evaluation mechanism, and the procedures to be followed.

### Establishing Initial Implementation Sites

Initial implementation sites (administrative districts) were selected according to the following criteria: presence of a functioning ARI and/or IMCI programme; commitment of the District Health Officer to the CLHP; availability of a designated health worker who, in addition to normal activities, assumed responsibility for implementation; and a catchment area of approximately 100,000 population. A plan for regular monitoring was then developed.

### Training Health Management Personnel

The programme manager and staff at each implementation site participated in intensive training to enhance their management skills and the computer skills needed to efficiently manage information.

### Training Inpatient and Outpatient Health Care Workers in Case Management

A CLHP training manual and modules were adapted to local conditions and used as a standard operating and reference manual. The training curriculum for health care workers, although focused on SCM of childhood lung diseases (pneumonia, tuberculosis, asthma, and HIV-related lung disease), also included case management of other major childhood illnesses (malnutrition, diarrhoea, malaria, anaemia, and meningitis) because children frequently presented with comorbid conditions. Annual training courses focused on theoretical and practical case management using local paediatric facilities and initially relied on an international course faculty. Local faculty gradually took on more responsibility for these courses. One-day follow-up training sessions took place 4–6 wk after the annual course plus ongoing in-service training.

### Monitoring Progress

Information routinely collected to provide patient care formed the core of the CLHP monitoring activities. Information was gathered using standard forms [12]. These documents were simple, clear, and kept to the absolute minimum required for adequate patient care and for monitoring and evaluating the CLHP. Monthly reports were generated on the number of pneumonia admissions by age and severity, treatment outcomes by age, and severity and monthly supply requirements.

This information was used for managing materials, for monitoring the quality of care, and for epidemiological surveillance. It was also used to assess health service utilization, the efficiency of the services provided, and the transparency of management procedures.

### Focusing Services on Vulnerable Children

The target group of the CLHP was vulnerable children with severe/very severe pneumonia who were at greatest risk of dying and who required hospital care.

### Managing Supplies and Materials

Uninterrupted supply of appropriate antibiotics was assured by using the routine reports to complete order forms to obtain annual supplies. To avoid “rupture of supply,” there was a “reserve” stock of medications (equivalent to 100% of the previous year's order) within the country. The information system was crucial for accurate planning of drug requirements.

### Evaluating the Services

The monthly reports on diagnosis, treatment, and outcomes provided the information needed to monitor the CLHP. They were also used to evaluate output and to advocate for resources. Systematic review identified and corrected problems in implementation. Completeness of the records was routinely evaluated.

Technical support by external experts twice a year focused on maintaining quality. Unit managers also met regularly to review their work and their problems. In addition, an independent review undertaken during the third year of the implementation period provided in-depth evaluation of the services and of the role of all the collaborators in the agreement.

### Scaling Up

Expansion was designed to achieve the widest possible access to the CLHP in the shortest time whilst maintaining quality. Five district/central hospitals were enrolled in October 2000 with five, six, and eight more enrolling in each subsequent year. By the end of the fourth year, all 22 district hospitals and two of the four central hospitals in Malawi had implemented the CLHP. Another central hospital was enrolled in the programme in 2004.

See Text S1 for further discussion on the framework and why this approach was chosen.

### Results Achieved

From October 2000 to December 2005, 312 health workers (approximately one-quarter of all nurses, clinical officers, and medical assistants working in government hospitals) participated in 5-d training courses.

- The 22 district and three central hospitals reported 48,702 cases between 1 October 2000 and 31 December 2005. The annual number of pneumonia admissions increased from 3,673 in the first full year (2001) to 7,516, 13,149, 10,134, and 13,674 in the subsequent years. Although this annual increase was partly due to the addition of new sites, for individual districts the mean yearly increase of admissions varied between 61.8% and 68.6%. The most likely explanation for this increase is that increasing confidence of the health care workers and community members in the CLHP resulted in more children being brought for treatment.
- The average cost per case managed in hospital was US$136 of which the investment cost for the international donor was US$8.34 per case.
- 10% of children treated were aged <2 mo, 52% were aged 2–11 mo, and 37% were aged 12–59 mo.
- In children aged 12–59 mo, 2% of cases were nonsevere pneumonia, 62.8% were severe pneumonia, and 25.2% were very severe pneumonia; in infants, 10.0% of cases had severe/very severe pneumonia.
- The proportion of children dying of pneumonia fell from 18.6% to 8.4%, a reduction of 54.8% over the baseline.

## Challenges

The major challenge facing the implementation of the CLHP in Malawi has been a shortage of health care workers. In particular, the attrition rate of trained workers has been high because of recruitment to the private sector, transfer of trained staff to other government hospitals, and deaths from HIV/AIDS. To address this problem, regular in-service and on-the-job training has been introduced and participation in the annual CLHP training course extended. The MOH has also increased the intake to nurses' training institutes and restarted the Medical Assistant training programme.

A second challenge has been continuing high case fatality rates in some districts due to aggravating factors such as malaria, malnutrition, anaemia, and HIV/AIDS. Case management of these conditions has now been introduced into the CLHP training course.

A third challenge has been a lack of equipment. To deal with this challenge, the CLHP has funded vehicles, computers, and other electronic equipment to improve supervision and communication. Oxygen concentrators have been provided at each site [13].

A final challenge facing the CLHP has been sustaining the programme beyond the cycle of foreign assistance. Following the programme's success, the MOH has included the CLHP in the Essential Health Package, which is funded through the Sector Wide Approach [14]. The CLHP has now been maintained for 3 y since the end of external project funding and is currently being expanded to nongovernment hospitals [15].

## Conclusions

The implementation of the CLHP in Malawi demonstrates the feasibility and effectiveness of a model programme based on the principles of the successful model for tuberculosis control to reduce case fatality in children hospitalized for pneumonia within first-level referral hospitals. The experience shows that while external funding is required to introduce the CLHP, the other key elements of the model are also necessary. Although it has not been possible to compare this approach, which has a substantial vertical component, with a locally integrated approach, the experience in Malawi suggests that this model could help the world achieve Millennium Development Goal 4.

## Supporting Information

Text S1 Discussion on framework/why this approach was chosen.
(0.11 MB DOC)

## Abbreviations

CLHP: Child Lung Health Programme
IMCI: Integrated Management of Childhood Illness
SCM: standard case management
