# Supplementary material for: Development and Implementation of a National Programme for the Management of Severe and Very Severe Pneumonia in Children in Malawi
Source: PLoS Med. 2009 Nov 10;6(11):e1000137. doi: 10.1371/journal.pmed.1000137 (PMC2766047; doi:10.1371/journal.pmed.1000137)
Supplement: Text S1 — Discussion on framework/why this approach was chosen. (0.11 MB DOC) [file pmed.1000137.s001.doc]

**Text S1**

1. **Discussion on framework/why this approach was chosen**

The MOH adopted the policies of the WHO/UNICEF-promoted programme on Integrated Management of Childhood Illness (IMCI) in 1992. In 2000 only 4 districts – those with the highest Infant Mortality Rate (IMR) in the country were in the process of implementing IMCI. The focus of the IMCI programme is the integrated case management of sick children at outpatient services from district hospital outpatient departments up to the most peripheral health post. At the time the IMCI programme did not include the care of referred children for further evaluation (chronic cough, persistent fever, wheeze) or for admission into hospital (severely ill children).

Since 1985 there had been an upsurge both in numbers and notification of TB cases and an increase in HIV related morbidity and mortality. The increased load on health care services and hospital capacity was of major concern.

The economic situation at this time made it difficult for the government of Malawi (GOM) to increase its health care expenditure to match the population growth and increased disease burden as described above and at the same time to sustain health programmes whose strategic components depended on the one hand on imported materials (drugs and laboratory supplies), and on the other hand on activities that required regular supervision.

In 2000 the main causes of morbidity and mortality in children <5 years of age were malaria, pneumonia and anaemia. Endemic malnutrition contributed largely to both morbidity and mortality. The management of sever and very severe pneumonia at inpatient level was not following international guidelines for developing countries.

There was no severity classification (standardized case definition) of pneumonia and no clear policy for the use of second line antibiotic regimens for treatment of the different categories. Oxygen was not available in most paediatric wards at district hospitals. The clinical overlap between pneumonia and malaria was problematic as the use of blood film examination for malaria parasites was infrequently used to clarify diagnosis. The HIV status of almost all children admitted with acute or chronic respiratory problems into district hospitals was unknown.

Less than 10% of staff involved in the care of sick children at the District Hospital level had been trained in standard case management of acute respiratory illness. Past child health training initiatives without follow up supervision have been largely ineffective at changing case management practices. Supervision was carried out very infrequently due to budgetary and other practical problems. Maternal education was inadequate.

There was a major problem identified with shortages of antibiotics at the district level mainly due to Central Medical Stores stock-outs.

The existing information system (tally card records) of Outpatient Department attendances and inpatient registrations were not sufficiently detailed or complete for the purposes of the CLH project. Also there were no standard MOH patient records at inpatient level.

Box 1. Main findings of situation analysis

| **Macroeconomic Situation**:   - Percent of population living in poverty 65.3% - GDP per capita $160 - Economically in the lower quartile of Southern African Development Community (SADC) - Contributing factors, floods, drought, global and others   **Demographics/health indicators:**   - Life Expectancy at birth   Male 41.4 years  Female 44.6 years   - Infant Mortality Rate (<1 year) 134/1000 live births - Child Mortality Rate (<5 years) 234/1000 live births - Maternal Mortality Ratio: 1120/100,000 live births - NACP estimates 14% of population are HIV+ rates higher in the urban areas (26%) - HIV sero-positivity in pregnant women varied from 10% in rural areas, close to 30% in urban areas - Upsurge in numbers and notification of TB cases - increases in smear negative and extra pulmonary TB - cure rates had fallen from 80% to 60%   **Main causes of morbidity/mortality in children <5 years of age:**   - Malaria +/- anaemia - Pneumonia – hospital case fatality rate > 26% - Pneumonia + malaria - Severe malnutrition (50% stunted, 4% severe malnutrition) - Gastroenteritis   **Health services:**   - Chronic under-funding - Only 1 MOH hospital per district regardless of population size - Doctor/population 1/110000 - Acute shortage of all health personnel - Very few staff involved in the care of sick children at district hospital level – at 1 hospital 0.5 nurse to >80 patients - Less than 6% staff trained in ARI standard case management - Chronic shortage of drugs/supplies at central level - No regular supply of oxygen available on paediatric wards - No regular supervision from central level - No patient records kept or regular reporting system in place |
| --- |

1. **Recording and reporting**

An Inpatient Recording Form originally developed primarily as an information collection tool. This underwent many revisions over the early months of 2000 and was then piloted in one of the DHs. The transition from being merely a data collection tool to a “patient treatment record” was at the request of the CLH Programme Manager, as at the time many of the DHs did not have any generic type forms to record ongoing treatment for inpatients. The form is constructed in such a way that the majority of information gathered only requires a tick so reducing the time required to complete it.

The information routinely collected formed the basis for materials management, in that supplies were ordered based on the needs reflected in the routine reports of activities. Besides providing the data for the planning of material requirements it also provided important information on quality of patient care and epidemiological surveillance. The extent of the problem (health services utilization), the efficiency of the services provided and the transparency of the management procedures was also derived from the data. This assisted the programme managers in annual planning discussions at which priorities for government spending are established and advocacy within the Ministry of Health and other governmental ministries.

Successful implementation of an information system required that indicators be identified, that they be routinely evaluated, and that the recording and frequency of reporting be optimal. Well defined epidemiological and operational indicators were established for measuring targets that were deemed measurable, valid, reliable and readily interpretable. The indicators used were: 1) type of patient, 2) type of treatment and 3) treatment outcomes.

Each district hospital CLHP Coordinator was expected to complete monthly reports on cases and treatment outcomes and submit to central level management unit. To assist with this activity and reduce amount of time required (average time 1-1.5 hrs/month) Epi-Info software was loaded onto each district hospital’s computer, where possible, for use by the CLHP designated focal person who attended workshops on how to use these programmes prior to their installation.

Systematic computer data entry and tabulation were essential for the regular accounting of services and supplies and generating data to feed back to the district hospitals and to the MOH. As of December 2005 2260 of the 2274 (99.4%) expected reports had been submitted.

The CLHP Coordinators analyse their own data and present their findings regularly at peer review meetings. A number have carried out operational research with the assistance of Central Level staff.

At Central Level a form for entering and checking data accuracy was developed on Excel by which to detect and correct mistakes.

The information system allowed close monitoring of activities, evaluation of outcomes and regular estimates of the needs and consumption of oral drugs as well as supplies for parenteral medications.

At the beginning of the programme there was already a data entry/management clerk at Central Level who entered the monthly reports into a Central Level computer. Analysis of this information allowed close monitoring of activities, evaluation of outcomes and regular estimates of the needs and consumption of oral drugs as well as supplies for parenteral medications.

To address the issue of rupture in drug supplies the CLHP maintained a "reserve" stock of medications (100%) within the health services system. In this way, every patient could be assured of receiving all the medications necessary for the treatment of pneumonia. Also the numbers of cases of pneumonia presenting for treatment showed a marked seasonality making it even more important to maintain a 12 month "reserve" at Central Level to control for varying levels of disease throughout the year. By implementing this system no rupture in supplies at the national level occurred during the first five years of operation. The data is also integrated into the HMIS system.

1. **Costs**

The MOH services are entirely financed by the government and external donors. The latter provide most of the development expenditure and finance a large percentage of the recurrent costs of preventive and promotive services. Free medical care for children less than 5 years of age in government-run facilities is the policy throughout the country.

Being part of the existing health system^6^ the national Ministry of Health (MOH) contributed 69.0% of the running costs comprising facilities and human resources. The donor (The Bill and Melinda Gates Foundation) provided 31.0% of the costs (a total of US$1.93 million over a five-year period) of which 21% was investment and 79% operating costs. See Figures 1 and 2 below.

**Sustaining the programme beyond the cycle of foreign assistance**

At the end of the Gates funding cycle the MOH included the CLHP in the Essential Health Package (EHP) which is funded through the Sector Wide Approach (SWAp).[[1]](#endnote-2) The CLHP Management Team worked with the DHOs to identify what activities needed to be included within the SWAp budget to maintain CLH services. According to the MOH annual report for the work of the Malawi health sector for the period July 2007 to June 2008 the ARI/CLH programme has continued functioning successfully with a further decrease in pneumonia CFR reported at 6.3 for year under review. It is now being expanded to non-government hospitals. [[2]](#endnote-3)

Figure 1 Breakdown MOH contribution

Figure 2. Breakdown of Gates funding expenditures

1. **The increase in number of admissions**

The fall-off of admissions in 2004 was due to poor central level supervision of programme during this period. The aggregate data does reflect the addition of new districts but when individual district admissions is analysed it demonstrates the following

**Table 1 Percentage increase in the number of pneumonia cases per district when compared to the first complete year of data collection.**

| **Districts** |  | **2001** | **2002** | **2003** |
| --- | --- | --- | --- | --- |
|  |  |  |  |  |
| Dedza |  | 100 | 202.6 | 248.5 |
| Mulange |  | 100 | 44.1 | 76.4 |
| Nkhata Bay |  | 100 | -22.2 | -33.2 |
| Ntcheu |  | 100 | 71.7 | 60.0 |
| Thyolo |  | 100 | 46.9 | 68.9 |
| Balaka |  |  | 100 | 25.2 |
| Kasungu |  |  | 100 | 23.2 |
| Machinga |  |  | 100 | 12.3 |
| Rumphi |  |  | 100 | -7.0 |
| Salima |  |  | 100 | 144.0 |
| Average increase | | | 68.6 | 61.8 |
| Max increase | | | 202.6 | 248.5 |

The first complete year is used as the basis to ensure that other factors such as season or time of year do not confuse the calculation. The mean increase per annum varied between 61.8% and 68.6%.

**4 Case management training**

The course focused on practical case management which was taught by using patients in clinics and hospitals. The philosophy used was one of knowledge transfer with the international course faculty progressively presenting less lectures and training sessions and the Malawi faculty taking on more and more responsibility for the course.

The implementation started in each district with the training of 10 staff members. These were divided into 2 groups and each group attends a 5-day course (40 hours) on Management of Childhood Lung Disease at District Hospital, using manuals and training materials developed by the Union in collaboration with local experts to teach the standard case management of major childhood lung disease, especially severe and very severe pneumonia. The courses were held at Lilongwe Central Hospital where the pediatric wards have a large number of children for the clinical practice sessions. The course agenda included clinical inter-active lectures, bedside clinical teaching, video clinical exercises, drills, clinical practice and exercises on the recording and reporting system. Although the course is entitled ”child lung health”, the curriculum includes the management of co-morbid conditions malaria, anaemia, measles, meningitis and severe malnutrition in relation to a child presenting with cough or difficult breathing, and also general pediatric issues such as triage of sick children, differential diagnosis of common problems, monitoring of sick children, discharge procedures and communication with parents.

During each course the participants were divided into three groups with an even distribution of members according to district, cadre and gender. These groups were formed for both clinical sessions and classroom group discussions.

The groups rotated through the three clinical areas i.e. inpatient, outpatient and health centre sessions. The clinical sessions were for 3 hours. The 2 groups in the hospital changed from inpatient to outpatient, and vice versa, halfway through sessions. The sessions provided adequate numbers of patients for the participants to assess, classify and identify correct treatment.

The MOH and DHO now provide funding within their SWAp budgets to continue with the annual training, as well as providing ongoing inservice training

1. Department of Planning, Ministry of Health. A joint programme of work for a health sector wide approach (SWAp) 2004-2010. Lilongwe Malawi: Republic of Malawi; March 2004. [↑](#endnote-ref-2)
2. Ministry of Health. Annual report for the work of the Malawi health sector for the period July 2007 to June 2008. Ministry of Health, Malawi. September 2008 [↑](#endnote-ref-3)
